# Towards practical object detection for weed spraying in precision agriculture

**DOI:** 10.3389/fpls.2023.1183277

**Published:** 2023-11-03

**Authors:** Madeleine Darbyshire, Adrian Salazar-Gomez, Junfeng Gao, Elizabeth I. Sklar, Simon Parsons

**Affiliations:** ^1^ School of Computer Science, University of Lincoln, Lincoln, United Kingdom; ^2^ Lincoln Centre for Autonomous Systems, University of Lincoln, Lincoln, United Kingdom; ^3^ Lincoln Institute of Agri-food Technology, University of Lincoln, Lincoln, United Kingdom

**Keywords:** automated weeding, computer vision, weed detection, object detection, dataset, precision spraying, spot spraying

## Abstract

Weeds pose a persistent threat to farmers’ yields, but conventional methods for controlling weed populations, like herbicide spraying, pose a risk to the surrounding ecosystems. Precision spraying aims to reduce harms to the surrounding environment by targeting only the weeds rather than spraying the entire field with herbicide. Such an approach requires weeds to first be detected. With the advent of convolutional neural networks, there has been significant research trialing such technologies on datasets of weeds and crops. However, the evaluation of the performance of these approaches has often been limited to the standard machine learning metrics. This paper aims to assess the feasibility of precision spraying via a comprehensive evaluation of weed detection and spraying accuracy using two separate datasets, different image resolutions, and several state-of-the-art object detection algorithms. A simplified model of precision spraying is proposed to compare the performance of different detection algorithms while varying the precision of the spray nozzles. The key performance indicators in precision spraying that this study focuses on are a high weed hit rate and a reduction in herbicide usage. This paper introduces two metrics, namely, weed coverage rate and area sprayed, to capture these aspects of the real-world performance of precision spraying and demonstrates their utility through experimental results. Using these metrics to calculate the spraying performance, it was found that 93% of weeds could be sprayed by spraying just 30% of the area using state-of-the-art vision methods to identify weeds.

## 1 Introduction

The requirement to feed an increasing global population means that effective methods to control weed populations and protect yields are as important as ever. However, effective weed control is becoming more challenging as climate change and intensification increase the competitiveness of weeds (Storkey et al., 2021). The mainstream approach to weed control is broadcast spraying, which involves spraying the entire field with a selective herbicide. This approach leads to significant wastage because much of the herbicide is applied to crops and bare soil rather than weeds. Additionally, the excessive use of herbicide increases harm to the surrounding ecosystems through leaching. An alternative approach, known as precision spraying, aims to target only the weeds with herbicide by first detecting them. Such an approach could have both environmental and economic benefits.

There have been numerous attempts to apply computer vision to the problem of weed detection. Most took a standard approach of collecting and annotating datasets, training detection algorithms, and evaluating the accuracy of crop and weed detection using standard machine learning (ML) metrics like mean average precision (*m*AP). These metrics are important when comparing detection approaches, but they do not offer much insight into whether the accuracy is sufficient to make precision spraying feasible. In order for precision spraying to be a feasible replacement for broadcast spraying, precision spraying must achieve a hit rate close to that of broadcast spraying—around 98%. In addition, precision spraying must result in a significant reduction in herbicide use. Lastly, detection must be fast enough such that weeds can be detected and sprayed in a single pass of the field without a significant reduction in vehicle speed compared to broadcast spraying.

This study aims to account for these three feasibility factors using three additional metrics. This paper introduces a new metric weed coverage rate (WCR), which identifies the proportion of weeds in the dataset that fall within the target area of a sprayer given a set of detections. While WCR is influenced by the accuracy of the model, it accounts for the precision of the spray nozzles and captures the interaction between these two factors. This gives insight into whether a detection strategy and nozzle configuration could achieve a suitable hit rate. Note that here we consider nozzle configuration at an abstract level; a detailed investigation of specific nozzle designs and performance is beyond the scope of this paper.

Moreover, we propose area sprayed, which represents the proportion of the area in the dataset that would be targeted for a particular detector and nozzle precision. This indicates whether a detector and nozzle configuration would result in a reduction of the area sprayed with herbicide. This metric is a proxy for herbicide usage and provides some insight into the wastage that any particular approach might incur. Lastly, inference speed is measured to determine whether detection is fast enough, using a single graphics processing unit (GPU), to facilitate real-time detection and spraying. Due to on-vehicle power constraints, detection methods need to be fast and compute efficiently as it is not possible to simply introduce more GPUs to overcome inference speed limitations.

The contribution of this paper is to establish the feasibility of precision spraying by evaluating a variety of deep learning-based vision methods applied to weed detection, not only using standard ML metrics, like *m*AP and inference speed, but also task-specific metrics as introduced in this paper: WCR and area sprayed.

Section 2 reviews the literature on weed detection and automated weeding. Section 3 describes the methodology, and Section 4 outlines the experiments conducted as well as presents the results and some analysis of the results. Section 6 considers the implications of the findings, and Section 7 concludes the paper.

## 2 Related work

Approaches to weed detection have evolved over the last two decades. Early approaches to detecting weeds relied on hand-crafted features to classify weeds based on color, shape, and texture features—for example, Nguyen Thanh Le et al. (2019) used local binary patterns with support vector machines for plant discrimination. While this approach only requires a small dataset for development, it may fail to generalize under varying light conditions.

Deep learning-based approaches were first implemented in work such as those of dos Santos Ferreira et al. (2017), which classified images containing weeds, and Dyrmann et al. (2016), which performed pixelwise segmentation of images of crops and weeds. These approaches proved more robust to variations in lighting conditions than the hand-crafted feature-based approaches. This work was followed by object detection-based approaches using popular object detection algorithms such as Faster-RCNN (Adhikari et al., 2019), YOLOv3 (Gao et al., 2020) and CenterNet (Jin et al., 2021). Other works have sought to improve the accuracy of deep learning-based weed detection. In Dang et al. (2023), an extensive range of Yolo models were tested to detect weeds in cotton crops, and data augmentation was shown to improve accuracy. In Wang et al. (2022), attention mechanisms were utilized to improve detection accuracy. In these studies, the results were evaluated using standard metrics such as *m*AP and inference speed. These metrics are useful in that they enable a comparison between detectors, but they do not provide an insight into the efficacy of the weed management.

Following improvements in weed detection technology, robotic weed control systems that enable precision weed management have been studied. “Spot” or “selective” spraying, the application case in this paper, involves switching an individual nozzle on or off to deliver chemicals only to the weeds (Hussain et al., 2020; Hussain et al., 2021). However, there is also work evaluating the efficacy of non-chemical systems that rely on mechanical tools (Wu et al., 2020) and laser-based tools (Mathiassen et al., 2006).

A variety of different approaches have been taken to trialing spraying systems. In Tufail et al. (2021), a detection system is deployed onto a Raspberry Pi and a spraying system is proposed, but the spraying performance of the system is not evaluating. In Ruigrok et al. (2020), YOLOv3 was integrated into a spraying system, and the hit rate was assessed in sugar beet and potato fields by evaluating the location of wet patches in the soil left by the sprayer. Similarly, Li et al. (2022) integrated YOLOv5 detection into a spraying system and assessed the hit rate using water-sensitive paper. These approaches provide an insight into the weed hit rat given a particular detector and a spraying system in a particular location. A similar approach was taken in Farooque et al. (2023), but they also published data on the herbicide savings made during their field tests. Their results showed 50% savings in herbicide use compared to broadcast spraying. In Zheng et al. (2023), the weed killing rate of the proposed system was compared with a broadcast approach as well as the herbicide saving.

These field trials reveal that the physical configuration of the system and the accuracy of the detection affect the overall precision of spraying. However, it is challenging to conduct field trials using a variety of prototype systems. The study described in this paper proposes a model to assess the suitability of different deep learning-based object detectors by taking into account different nozzle precisions and different field environments. The evaluation of each approach will balance optimal inference speed, weed coverage rate, and area sprayed for a weed sprayer in a field environment, and this assessment will be contrasted with an evaluation using standard ML metrics. In this way, the proposed approach will estimate the spraying performance of several detectors and nozzle configurations like a field trial, but with the reproducibility of studies using datasets.

## 3 Methodology

**Figure 1:**
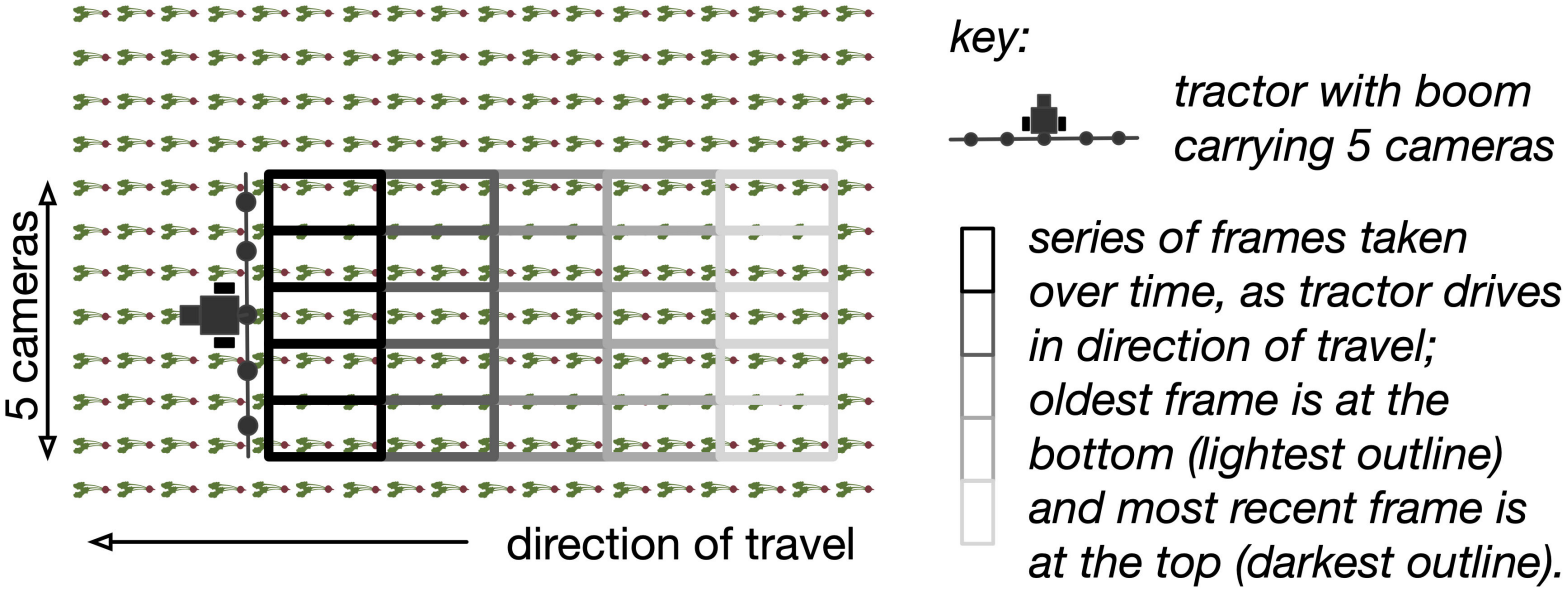
A top-down view of a small precision sprayer. The boom carries five cameras, and the nozzles would be positioned along the boom as well. Each camera captures images as the vehicle over the field in the direction of travel indicated. Each row of images represents the set captured by the cameras at the same point in time; each column represents the passage of time as the tractor drives. The images here do not overlap.

The scenario that this paper considers is shown in **Figure 1**. This consists of a sprayer boom, equipped with cameras, fixed a short distance ahead of nozzles that dispense herbicide, and mounted on the back of a farm vehicle. This enables the cameras to process images of an area before the nozzles pass over that area. Depending on whether weeds are present in the image, nozzles can then be actuated as the corresponding region passes beneath the nozzles.

### 3.1 Detection accuracy evaluation

Object detection algorithms locate objects of interest within images and demarcate them using bounding boxes. Deep learning-based object detectors learn from a set of labeled training images how to locate objects within an image. The accuracy of a detector can then be determined by evaluating the detection performance on a set of labeled test images.

A conventional metric to evaluate the performance of object detectors is mean average precision (*m*AP). To calculate *m*AP, a threshold is first defined for the intersection over union (IoU) value, and this is used to distinguish between true positive (TP), false positive (FP), and false negative (FN) detections. IoU measures the accuracy of the predicted bounding box circumscribing the object identified and is equal to (*G* ∩ *D*)/(*G* ∪ *D*), where *G* is the area of the ground-truth (labeled) bounding box and *D* is the area of the bounding box predicted by the model.

Precision (*P*) is the proportion of correctly classified objects over all objects detected, TP/(TP + FP).

Recall (*R*) is the proportion of correctly classified objects over all labeled objects, TP/(TP + FN). These metrics are used to calculate average precision (AP):


$$\text{AP}=\sum_{n}(R_{n}-R_{n-1}).P_{n}$$

where *n* is the IoU threshold rank. Because AP is dependent on the IoU threshold value, mean AP or *m*AP averages the AP over several threshold values. This article uses the COCO standard (Lin et al., 2014) of 10 IoU thresholds between 0.05 and 0.95 that are 0.05 apart.

While *m*AP is a good measure of the performance of an object detector, it focuses on identifying the object very precisely. In practice, for a sprayer, the level of precision for spraying is limited by the spray nozzle, and as we will see, it is possible for an object detector that has a relatively low *m*AP to still be good enough to be effective in ensuring that weeds are covered with herbicide.

### 3.2 Spray precision evaluation

**Figure 2:**
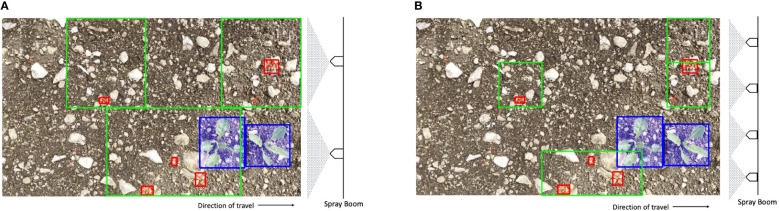
**(A)** Two nozzles. **(B)** Four nozzles. Example of how spraying areas are proposed with detected bounding boxes containing weeds *d_ij_* (red) and a detected beet (blue) for the same image and with two and four nozzles. The green boxes are derived from the union of the individual spraying areas *B_ij_* as a result of merging the overlapping spraying areas. The total area of all the green boxes in the images is *S_i_*.

In order to understand how the accuracy of a detector impacts the potential weed hit rate, a new metric was devised, called WCR. This estimates, given a particular number and density of nozzles, how many weeds would be hit if the detector aimed to hit all detected weeds. The metric is calculated using a simple model of spraying. Given the *i*-th image from a dataset with *I* images, a sprayer moving parallel to the width of the image (*W_i_*) would have a spray boom that runs parallel to the height of the image (*H_i_*) as illustrated in **Figure 2**.

The precision of the spray nozzles can vary from spraying an area 500 mm wide, used in broadcast spraying, to around 100 mm for precision spraying. This variation is dictated primarily by the nozzle type, although pump pressure has an impact on the area sprayed by the nozzle, too. In order to understand how varying the spray nozzle precision impacts the hit rate, given a set of detections, different nozzle configurations are modeled. This is shown in **Figure 2**. The image is split into *n* horizontal strips along its height, each with width *W_i_* pixels and height *H_i_*/*n* pixels, which represents the area of the image where a particular nozzle could spray. When *n* is one, the whole height of the image is targeted by one nozzle; by contrast, when *n* is four, a smaller section of the image is targeted by a more precise nozzle. Assuming a standard deployment camera, such as a RealSense D435i with a focal length of 1.93 mm and F-number of f/2.0, situated at a standard height from the canopy (∼500 mm), the image would cover an area of ∼670 mm by ∼380 mm. Since the spray boom is aligned along the shorter height of the image (*H_i_*) by varying the nozzles from one to four, the area that each nozzle covers is varied from 380 to 95 mm.

The *i*-th image in the dataset has *J_i_* detected weed bounding boxes, each with width *w_i,j_* and height *h_i,j_*. For each strip that intersects with a detected weed bounding box, a spray area *B_i,k_*, is proposed in that strip, where *B_i,k_* is the *k*-th spray area proposed in the *i*-th image. *B_i,k_* has the height of the strip, and its width is *max*(*H_i_*/*n,w_i,j_*). In the *i*-th image, there are *K_i_* of these spray areas proposed. To derive *S_i_*, which is the total spray area within the *i*-th image, to the exclusion of overlapping regions, the union of the proposed spray areas is calculated as follows:


(1) $$S_{i}=\overset{K_{i}}{\underset{k}{\cup }}B_{i,k}$$

where both *S_i_* and *B_i,k_* are measured as the number of pixels enclosed in the area. The generation of *S_i_* is illustrated in **Figure 2**.

The spray area is larger than just the bounding boxes because the size is partly determined by the width of the spray (compare **Figures 2A, B**). WCR differs from *m*AP because that additional area, while increasing the herbicide used and decreasing *m*AP, will sometimes “hit” weeds that have evaded detection. WCR is defined as follows. A weed is counted as having been sprayed if it is wholly contained in the spray area:


(2) $$\text{Sprayed}\left(G_{i,m}\right)=\{\begin{array}{l} 1,\text{if }G_{i,m}\subseteq S_{i}\\ 0,\text{otherwise} \end{array}$$

where *G_i,m_* is the area of the *m*-th ground truth weed bounding box in image *i*. Then, the WCR for a dataset is the fraction of the weeds that are counted as having been sprayed:


(3) $$\text{Weed coverage rate }\left(I\right)=\frac{\sum_{i\in I}\sum_{m\in M_{i}}\text{Sprayed}\left(G_{i,m}\right)}{\sum_{i\in I}M_{i}}\times 100$$

where *M_i_* is the total number of ground truth weed detections in the *i*-th image. In addition, we compare the area that has been sprayed with the total area of all the images in the dataset:


(4) $$\text{Area sprayed }\left(I\right)=\frac{\sum_{i}S_{i\in I}}{\sum_{i\in I}W_{i}\times H_{i}}\times 100$$

where the areas are measured in pixels. Since the area sprayed is a proportion of the total area that would be covered by broadcast spraying, the volume of herbicide saved is proportional to 100 – Area sprayed. These metrics measure the number of weeds that we spray and the amount of herbicide used. The higher the WCR, the more plants we hit, and the higher the area sprayed, the more herbicide is used. A farmer would typically want to maximize WCR while minimizing the area sprayed.

### 3.3 Inference speed evaluation

In order to establish the frame rate requirements, we need to make some assumptions about the design of a sprayer. The boom on a typical sprayer is 24 m, and the recommended height to operate the boom above the crop canopy is 500 mm. We established empirically that, at this height, a typical camera with a 1.8:1 aspect ratio can cover 670 mm × 380 mm. It is widely considered best practice to limit the application speed to around 15 mph or 6.7 m/s to prevent spray drift and ensure a consistent herbicide application. According to a recent survey, the 15 mph or 24.1 kmph limit is broadly adhered to by farmers (Virk and Prostko, 2022). With the long edge of the image aligned along the spray boom, we need 35 cameras, and each camera can cover 380 mm in the direction of travel. At 6.7 m/s, we will need to process 28 frames per second (assuming no overlap). Thus, across all 35 cameras, the required frame rate will be 980. With the short edge of the image aligned along the boom, we would need 64 cameras and process 16 frames a second per camera, giving a required frame rate of 1,024 frames per second (FPS).

## 4 Experiments

### 4.1 Datasets

Two sugar beet and weed datasets were used: the Lincoln beet (LB) dataset, which was annotated as part of this work, and the Belgium beet (BB) dataset, which is from Gao et al. (2020). The images contain pictures of sugar beet and weeds with their respective bounding box locations. The BB dataset contains 2,506 images of 1,800 × 1,200 pixels. The LB dataset consists of 4,405 images of 1,920 × 1,080 pixels.

Images in the LB dataset were extracted from videos taken in three different sugar beet fields. Frames were taken such that they were sufficiently far apart to avoid repetition in the dataset. Unlike the images in the BB dataset, which we all collected in a single day, the LB images were collected at different times over the crop’s early growing period. The data collection spanned May–June 2021, where each field was scanned, at minimum, on four different dates a week apart to record weeds at different stages of growth. For all the data collection sessions, the distance from the camera to the ground was approximately 500 mm. Two cameras were used: one with 12 megapixels, 26 mm focal length, and f1.6 aperture; the other with 64 megapixels, 29 mm focal length, and an aperture of f2.0. The original size of the pictures from both cameras was 2,160 × 3,840 pixels.

**Figure 3:**
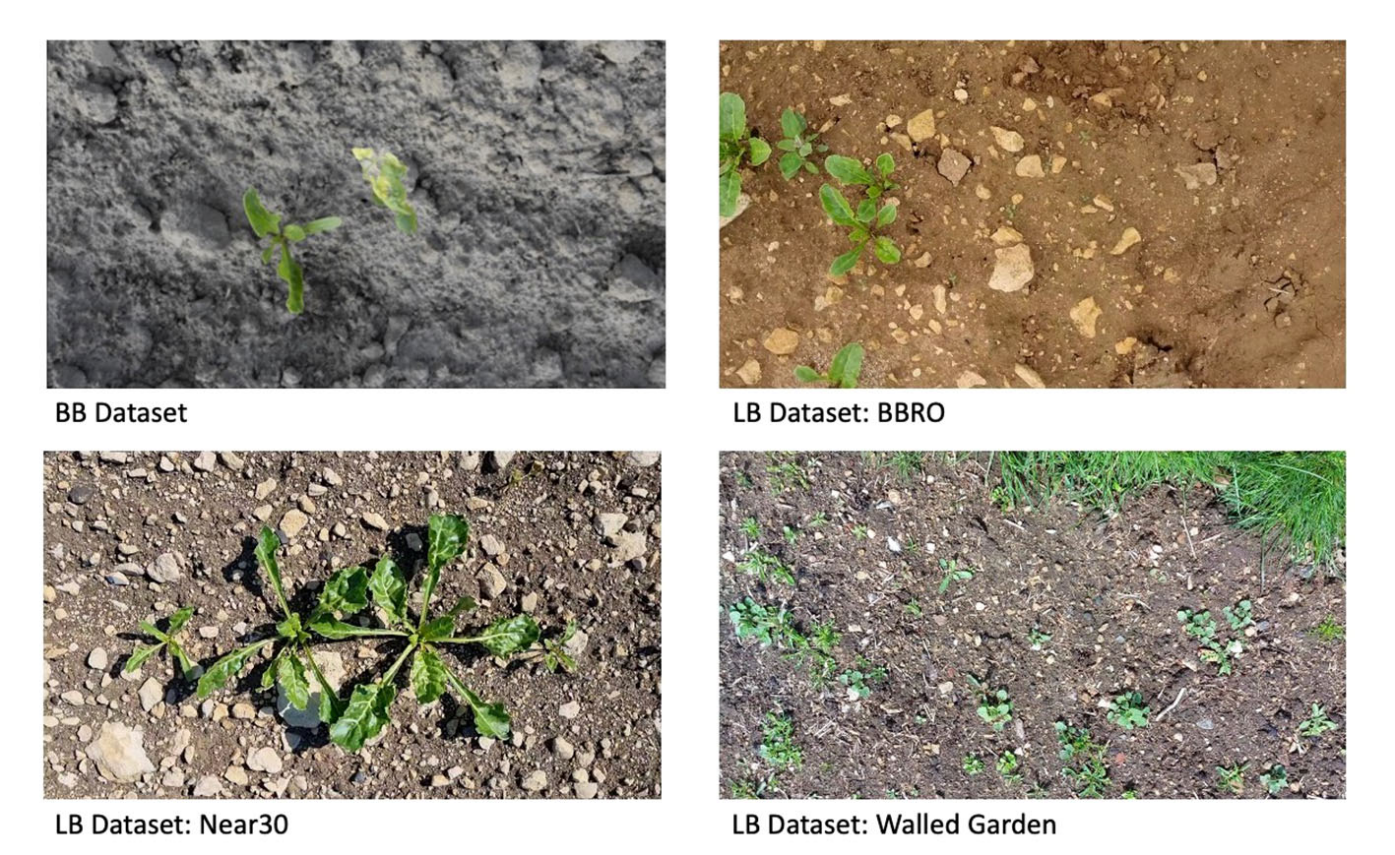
Examples from the Belgium beet dataset and the three different fields in the Lincoln beet dataset.

The fields used for the LB dataset are near Lincoln, UK. They are situated in different locations with varying conditions as to the type of soil, distribution of the plants, and weed varieties. Two of the fields, called the Near 30 and the BBRO, are typical of commercial sugar beet fields in the UK, while the third field, known as the walled garden, is a small plot in an enclosed brick-walled garden which is less representative of a commercial operation in the UK. In Gao et al. (2020), an overall *m*AP of 0.829 was achieved using the BB dataset. This was composed of a *m*AP of 0.761 for the weed class and 0.897 for the sugar beet class. **Figure 3** shows examples of the BB dataset and the three fields used to create the LB dataset.

Both datasets present different item distributions and visibilities. The BB dataset has a lower number of items per picture than the LB dataset. In terms of visibility, the items in the LB dataset are proportionally smaller than the items in the BB dataset, and the items in the BB dataset have higher levels of interitem occlusion. **Table 1** shows the visibility and distribution characteristics of both datasets and the characteristics of each of the items in the dataset.

**Table 1:** Characteristics of the Belgium beet (BB) and Lincoln beet (LB) datasets at dataset level (top) and at item level (bottom).

	BB dataset	LB dataset
Number of images	2,506	4,402
Number of items	5,578	39,246
Average items per picture	2.22	8.915
Average percentage of the bounding box that is occluded	0.02187	0.0176
Average area of the image occupied by bounding boxes	0.09883	0.0717
Number of sugar beets	2,654	16,399
Average sugar beet plants per picture	1.059	3.725
Number of weeds	2,924	22,847
Average weed plants per picture	1.166	5.190
Average area occluded in sugar beet bounding boxes	0.0239	0.0324
Average area occluded in weed bounding boxes	0.0159	0.001
Average image area occupied by a sugar beet bounding box	0.0899	0.033
Average image area occupied by a weed bounding box	0.0360	0.002

### 4.2 Data preparation

For the experiments, each dataset was randomly split into training, test, and validation sets with 70%, 20%, and 10% of the dataset images, respectively. For training and testing, we resized the images and labels in both datasets to make the large side of the image fit in a 640-pixel dimension while maintaining the width/height ratio of the raw images.

### 4.3 Models and parameters

For the identification of weeds, both one-stage detectors and two-stage detectors were implemented. Two-stage detectors tend to produce more cautious predictions than the one-stage detectors. By contrast, one-stage detectors tend to be faster. By implementing both approaches, it is possible to determine whether the speed of the one-stage detectors or the cautious predictions of the two-stage detectors would have any impact on the metrics used (Soviany and Ionescu, 2018). The one-stage models are Yolov5s, Yolov5m, Yolov5l, Yolov5x (Jocher et al., 2021), and Yolov3 (Redmon and Farhadi, 2018), where all models use Darknet-53 (Redmon and Farhadi, 2018) (DN-53) as a backbone. The two-stage detectors are based on Faster R-CNN (Ren et al., 2015) with three different backbones: one with a ResNet-50 backbone (He et al., 2016) and a Feature Pyramid Network (Lin et al., 2017) (FPN) neck—namely, ResNet-50-FPN (R-50-FPN); one with ResNet-101-FPN (He et al., 2016) (ResNet-101-FPN); and another detector with ResNeXt-101-FPN (Xie et al., 2017) (Rx-101-FPN).

During training, for the one- and two-stage models, the optimizer was stochastic gradient descent, the learning rate was 1 × 10^−2^, the momentum was 0.937, and the learning decay was 5 × 10^−4^. In both model varieties, the networks were pre-trained on the COCO dataset (Lin et al., 2014). For testing, the confidence threshold was 0.05, and the intersection over the union threshold was 0.5. These values are obtained from a grid search maximizing the *m*AP. For the sake of consistency, no data augmentation was applied in the training of these models, although using data augmentation may improve the accuracy. The models were trained and tested on a GTX2080Ti processor with 11 GB of VRAM.

For all detectors, the batch size (the number of images fed to the models simultaneously) used for training was the maximum number of images that can fit in the GPU’s VRAM, alongside the detector. The number of epochs for training was 300. For each model, the model weights used for testing were the ones with the highest *m*AP on the validation set during the training process.

## 5 Results

We evaluated the trained models in several ways across both the BB and LB datasets and report both *m*AP and inference speed as well as our new metrics: weed coverage rate and area sprayed.

### 5.1 Accuracy

**Figure 4:**
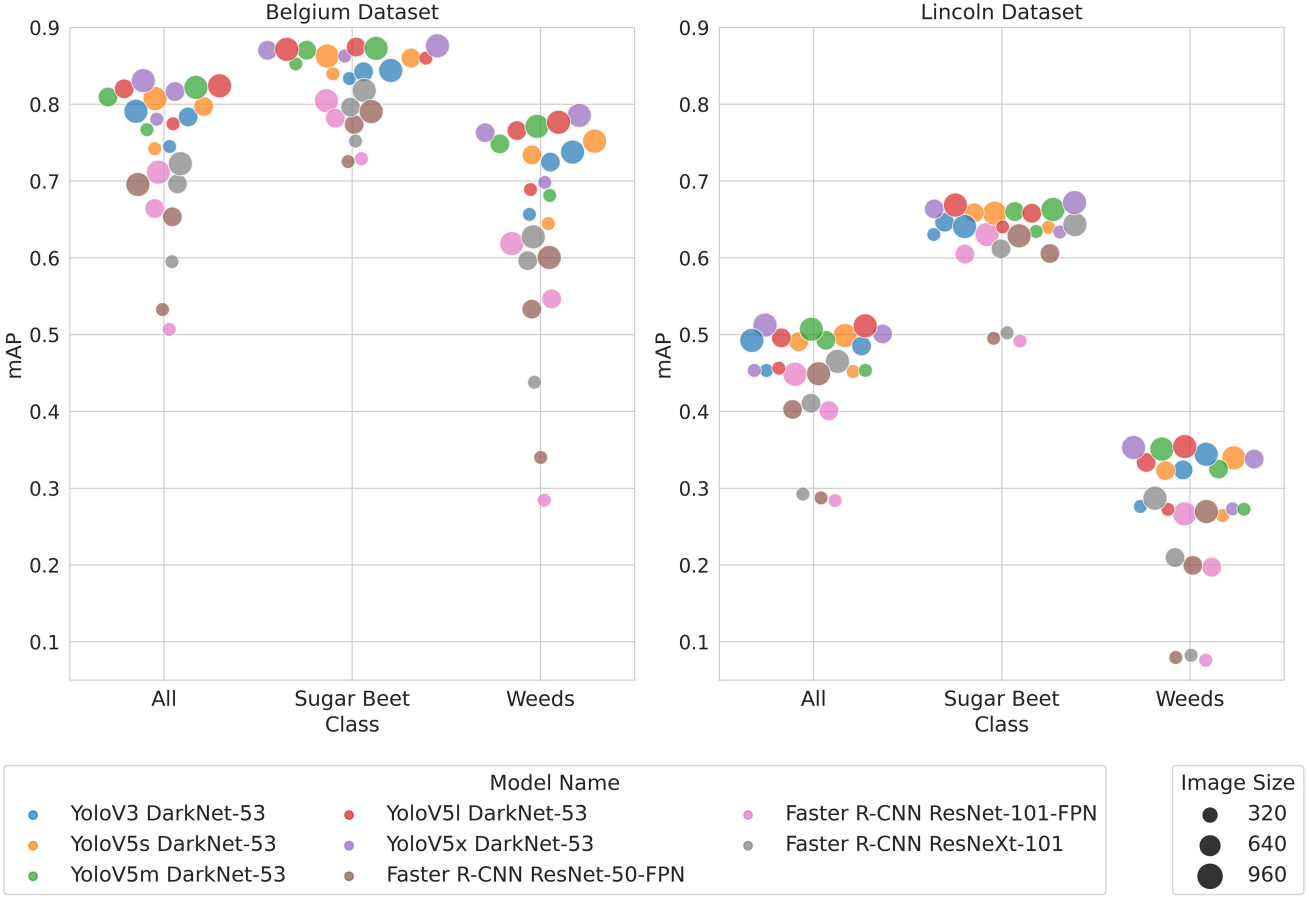
Swarm plots show a comparison of *m*AP between models. Small perturbations in the *x*-value have been applied so that all markers can be seen.

**Figure 4** provides a conventional evaluation giving *m*AP for each model and image size. The *m*AP for both classes in the LB dataset is lower than in BB. This could be because of the higher density and smaller size of the plants in the LB dataset as well as the greater diversity of plant sizes and field environments. **Figure 4** shows that the YoloV5 models typically achieved a higher *m*AP than the other models. YoloV3 performed comparably to YoloV5 on the LB dataset but less well on the BB dataset. The Faster R-CNN models produced a lower *m*AP than either the YoloV5 models or YoloV3. Reducing the resolution from 960 to 640 in the Yolo models had a negligible impact on *m*AP in most cases, while it had a bigger impact for the Faster R-CNN models. Similarly, reducing the resolution from 640 to 320 resulted in a slight reduction in *m*AP in the Yolo models but a much larger reduction in the Faster R-CNN models. The *m*AP for the weed class was lower than for the sugar beet class in both datasets. The difference in accuracy between classes is more significant in the LB dataset than in the BB dataset.

### 5.2 Accuracy at different growth stages

**Figure 5:**
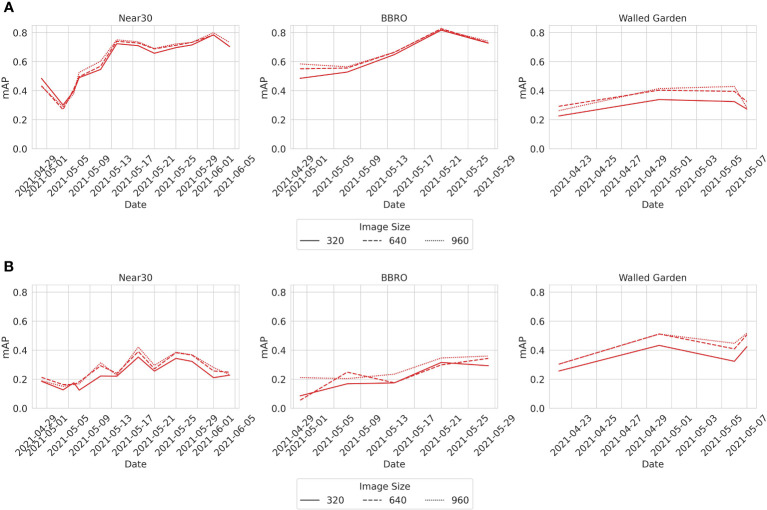
**(A)** Sugar beet. **(B)** Weeds. The *m*AP of the YoloV5l model at different collection dates from the three fields where data was collected—namely, the Near30, the BBRO, and the Walled Garden. The trend for other models is similar, and the plots for these models are available in the **Supplementary Material**.

The LB dataset provides metadata for the time the image was taken and in which field. From this, it is possible to analyze how the *m*AP changes as both the sugar beet and weeds grow. **Figure 5** shows the *m*AP changes over time in each of the fields where the images were taken. **Figure 5A** shows that the *m*AP clearly improved over time for the sugar beet class. The improvement is more marked initially possibly due to increases in plant size, which was eventually being offset by the increasing occlusion. **Figure 5B** shows that, for the weed class, there is a more subtle improvement over time and more variation in the *m*AP between collections. Similarly, in the weed class, the improvement plateaus suggest the benefits of increasing the plant size and the differentiation being offset by some occlusion. Lastly, it is interesting to note that all the models follow a similar trend with *m*AP falling and rising at the same points. This suggests that all the models are learning similar sets of features. Since image collection spanned a month during the early growth stages of the sugar beet, it should be noted that the collection ended before the canopy closure caused a significant occlusion. Therefore, these results only reflect changes in detection accuracy during the early growth stages of both weeds and sugar beet.

### 5.3 Speed

**Figure 6:**
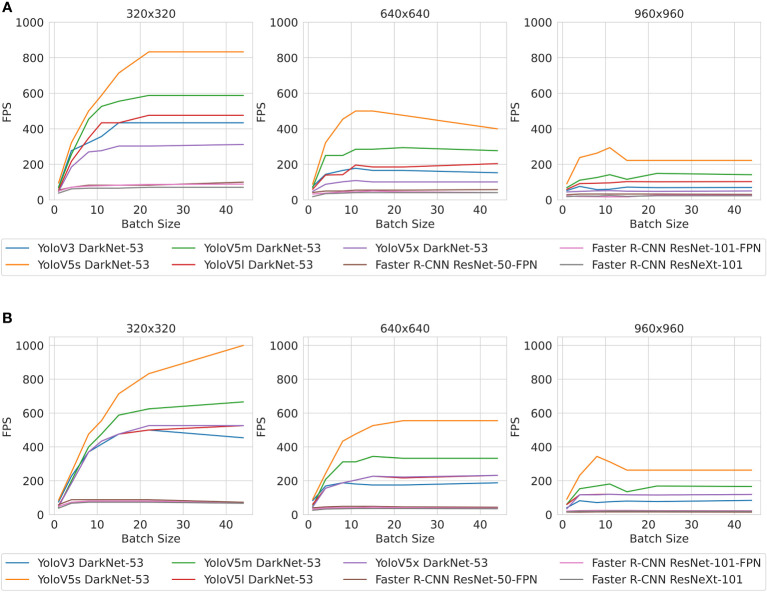
**(A)** Belgium beet dataset. **(B)** Lincoln beet dataset. Inference speed of models (in frames per second, fps) and image sizes.

**Figure 6** shows how inference speed (measured in FPS) changes over different batch sizes on each dataset. **Figure 6** shows that YoloV5s on 320 resolution has the fastest inference speed by a significant margin. All models ran faster with lower-resolution images, and models running inference on lower-resolution images saw much greater gains in speed as the batch size increased. In general, the Yolo models outperformed Faster R-CNN models, and the Yolo models showed greater improvements in inference speed as a result of reducing the resolution than Faster R-CNN models.

### 5.4 WCR and area sprayed

**Figure 7:**
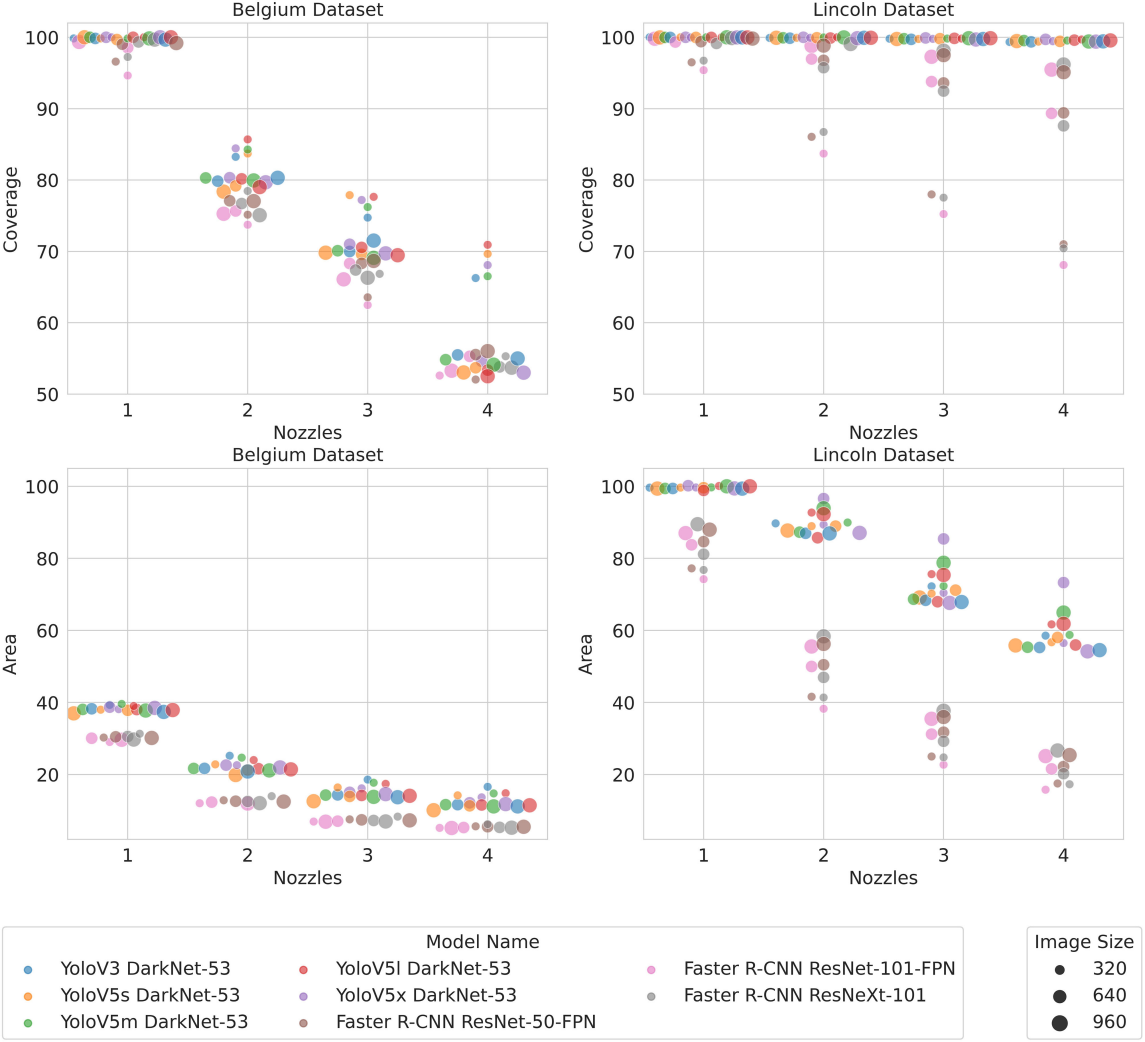
Coverage and area sprayed with different numbers of nozzles. Perturbations in the *x*-values have been added to make the markers more visible where they would otherwise overlap.

**Figure 7** reports the WCR and area sprayed for one to four nozzles. On the BB dataset, **Figure 7** shows a significant decrease in WCR when the density and precision of the nozzles increased. At each nozzle configuration, the Yolo models achieved a slightly higher WCR than the Faster R-CNN models. However, the Yolo models showed a small increase in area sprayed compared to Faster R-CNN models. Interestingly, the Yolo models with lower-resolution 320 × 320 images had a higher WCR than the other Yolo models despite achieving a lower *m*AP. This increased coverage achieved by lower-resolution Yolo models was only associated with a slight increase in area sprayed compared to the other Yolo models.

On the LB dataset, **Figure 7** shows that the coverage remained close to 100% for all the Yolo models, regardless of resolution, even as the precision and density of the nozzles increased. Faster R-CNN performed less well but still retained coverage above 85% for all nozzle configurations at 960 and 640 resolutions. Only Faster R-CNN models using 320 × 320 images saw a significant decrease in WCR as the nozzle precision and density increased. While Yolo and Faster R-CNN had a comparable coverage using higher-resolution images, the Yolo models sprayed a much larger area to achieve that level of coverage across nozzle configurations. Equally, while the coverage achieved by the Faster R-CNN models with low-resolution images is significantly lower than the other Faster R-CNN models, the area sprayed is quite comparable.

### 5.5 Comparison of weed coverage rate and area sprayed with *m*AP

**Figure 8:**
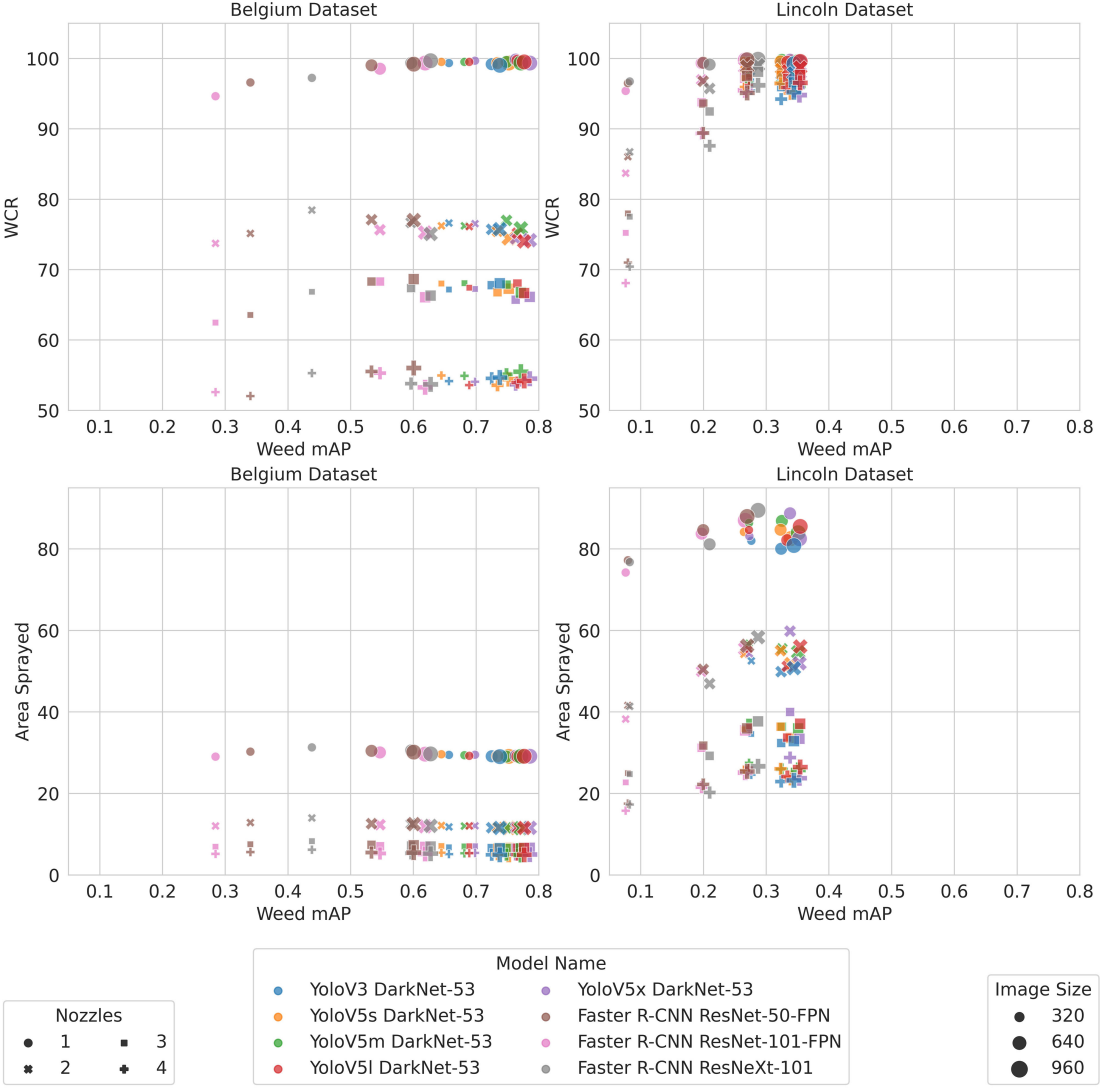
The top row shows the relationship between coverage and *m*AP, and the bottom row shows the relationship between the area sprayed and *m*AP.

The WCR and area sprayed results indicate that models with comparatively low *m*AP values can perform as well as models with higher *m*AP values. In order to better understand the relationship between WCR and area sprayed, **Figure 8** plots the WCR and area sprayed against *m*AP. **Figure 8** shows that, on the BB dataset, there is almost no correlation between *m*AP and coverage. WCR remains fairly constant even as *m*AP increased significantly. Similarly, there is no clear relationship between the area sprayed and *m*AP.

On the LB dataset, **Figure 8** shows that there is a weak correlation between WCR and *m*AP. However, as discussed in Section 5.4, the coverage is the same for higher-resolution Faster R-CNN models and Yolo models despite the *m*AP being different. On the LB dataset, **Figure 8** shows that the area and *m*AP are weakly positively correlated. This suggests that some models with a high *m*AP actually spray a greater area in some cases.

**Figure 9:**
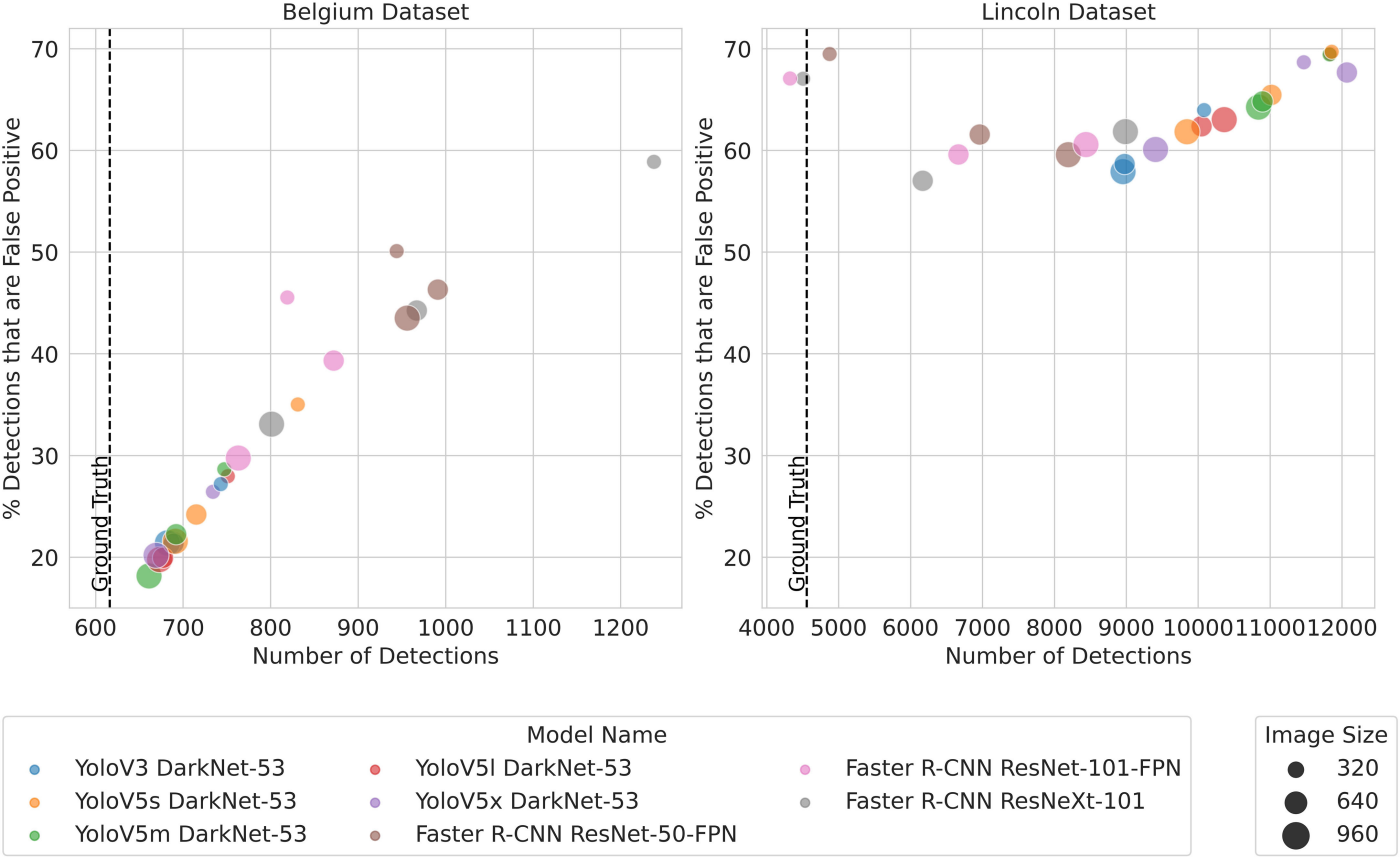
Number of detections from each model.

**Figure 10:**
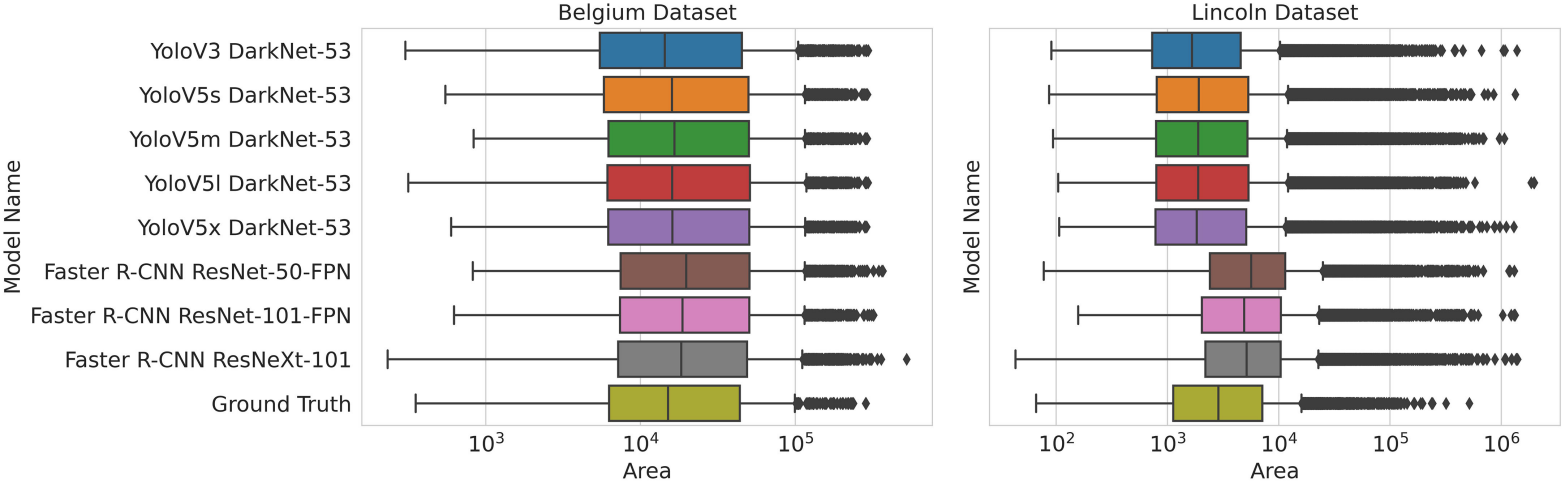
Distribution of bounding box areas for all models across resolutions. The width of the box goes from the lower to the upper quartile with the line through the middle of the box representing the median. The line through the box runs from the lower extreme to the upper extreme. Outliers are represented by the markers after the extremes.

In order to clarify why some models have a comparable WCR while the area sprayed varies, the quantity and area of predicted bounding boxes, which form the basis of these metrics, are plotted in **Figures 9** and **10**, respectively. **Figure 9** shows the number of detections plotted against the proportion of detections that are false positives, while **Figure 10** shows the distribution of areas of the predicted bounding boxes for each model.

For the BB dataset, the distribution is similar for all models, with Faster R-CNN bounding boxes only slightly larger on average. The distribution of bounding box areas also closely matches the areas of the ground truth bounding boxes. **Figure 9** shows that more bounding boxes are predicted for Faster R-CNN, but more of them are false positive. Therefore, Faster R-CNN predicts a greater quantity of larger bounding boxes, but the area sprayed is still low compared to Yolo. This suggests that Faster R-CNN predicts clusters of bounding boxes, more of which are false positive, compared to Yolo that predicts fewer bounding boxes that are more scattered with fewer false positives. This also explains why Faster R-CNN’s lower *m*AP is not reflected in coverage or area sprayed, as the extra false positives are closely clustered to true positives.

On the LB dataset, **Figure 9** shows that Yolo models produce far more detections than Faster R-CNN models. However, both models produced a large number of false positives relative to total detections.

When models produce a large number of detections, it can affect the inference speed. Non-maximum suppression is used to remove duplicate bounding boxes. Since it has a complexity of *O*(*n* ^2^), where *n* is the number of detections, models that produce a large number of detections can have slower inference speeds. This discrepancy is shown in **Figure 6**, where YoloV5s has a slower inference speed on the LB dataset compared with the BB dataset. This is explained by the difference in the number of detections YoloV5s produces on each dataset, as shown in **Figure 9**.

**Figure 10** shows that Faster R-CNN models produce larger bounding boxes on average than Yolo’s predicted bounding boxes or the ground truth bounding boxes. On average, Yolo models’ bounding boxes are slightly smaller than the ground truth bounding boxes. There are far more very large bounding boxes, represented as outliers, predicted by all models on the LB dataset compared to the BB dataset. While Faster R-CNN produces larger bounding boxes on average, this is not offset by the greater number of detections produced by Yolo models, most of which will be false positive, that account for the larger area sprayed. The large number of false positives as a proportion of detections accounts for the far lower *m*AP on the weed class on the LB dataset.

Overall, when comparing our new metrics to *m*AP, it is clear that our metrics elucidate factors in how the models detect weeds and how those detections will be used by a sprayer to spray herbicide—factors that are not accounted for in the *m*AP calculation.

## 6 Discussion

Initially, we evaluated our model according to standard *m*AP metrics and found that Yolo models were more accurate and faster than Faster R-CNN models. On the BB dataset, YoloV5l achieved a *m*AP of 94.0 and 82.8 on the sugar beet and weed class, respectively. This is compared to *m*AP of 0.897 and 0.761 for the sugar beet and weed class, respectively, in the previous work using this dataset (Gao et al., 2020). However, in this study, we wanted to examine these results in more depth and assess the practical feasibility of deep learning-based methods for precision spraying applications.

Firstly, we investigated how the accuracy of detection improved as plants matured through the early growth stage. Our results show that, in general, detection accuracy improves during the period captured in the LB dataset for both sugar beet and weeds.

Secondly, we investigated the inference speed of different models using images of different resolutions to verify whether inference could be performed fast enough for real-time precision spraying. Yolov5s with 320 × 320 images had the highest inference speed out of all the models tested, achieving 833 FPS on the BB dataset and 1,000 FPS on the LB dataset. This is in the right ballpark to achieve our target pace of 15 mph (6.7 m/s)^1^ that would make in-the-field spraying with YoloV5s using 320 × 320 feasible using GTX 2080Ti.

Lastly and most importantly, we propose the new metrics WCR and area sprayed that aim to directly measure the performance of herbicide spraying based on the detections from an object detector. Area sprayed followed the same trend of decreasing as the density and precision of nozzles increased in both the BB dataset and the LB dataset. By contrast, the WCR results were quite different with coverage being less affected by a change in nozzle configuration in the LB dataset than in the BB dataset. These differences are likely explained by the overall area sprayed being much greater in the LB dataset, leading to many more off-target hits.

When using the metrics to compare models, our results demonstrate the need to trade off WCR and area sprayed rather than select the model with the highest *m*AP—for example, Faster R-CNN had a lower area sprayed across nozzle configurations and datasets while mostly retaining a comparable WCR at higher resolutions despite having a lower *m*AP. A further analysis suggested that the lower area sprayed was a result of Faster R-CNN producing fewer false positive detections. These results suggest that increases in false positives that may not influence the *m*AP to a significant degree may still have a significant impact on herbicide usage. It follows that *m*AP alone does not provide the granularity to be certain that a detector is the optimal choice for weed detection in precision spraying. Overall, our results help illustrate that the choice of model is a trade-off between WCR, area sprayed, and inference speed.

A limitation of the results presented in this paper is that they were computed on non-contiguous images. This means that the entire spray area needs to fit within the image. However, if the images were contiguous, it would be possible to allow the spray area to run over into the next frame. Additionally, there are other factors that affect the hit rate that were not modeled here, such as spray drift and variation in nozzle actuation time. Modeling these aspects will be addressed in future work.

## 7 Conclusions and future work

This paper proposes a method for evaluating the feasibility of different state-of-the-art object detection methods applied to selective spraying.

The WCR metric demonstrates that many state-of-the-art object detectors can achieve a hit rate close to that of broadcast spraying; however, this is dependent on the precision of the nozzles in the spraying system as well as the field environment. The work shows that a high WCR needs to be balanced with other factors like inference speed and herbicide usage. Herbicide usage was proxied via the area sprayed metric which estimates the overall area to which herbicide would be applied by targeting the detections. This metric highlights options that produce clear reduction in area sprayed and, hence, herbicide required. As with many multi-criteria optimization problems, there is no single clear winner; however, these metrics help highlight the advantages and drawbacks of different approaches, demonstrating that when it comes to practical deployment, it is not just about *m*AP.

The method proposed in this work gives an insight into how detection accuracy and nozzle configuration interact to affect spraying performance. In practice, these metrics could be used to find the optimal physical configuration and detector for a precision sprayer used on a particular crop. One limitation is that there may be other factors that influence weed coverage and area sprayed that this method does not account for.

Future work will aim to improve the frame rate achievable on a single GPU. Additionally, in order to make WCR and area sprayed estimations better reflect real-world spray accuracy, contiguous images will be used and more properties of the sprayer will be accounted for, including nozzle response times and spray drift. In this work, a single crop is used, so an extension of this work may cover a variety of crops. Lastly, a prototype precision sprayer will be used to measure WCR and area sprayed in real field environments. These real-world measurements could then be compared to the values calculated using the proposed method.

## Data availability statement

The datasets presented in this study can be found in online repositories. The name of the repository and accession number can be found below: https://github.com/LAR/lincolnbeet_dataset.

## Author contributions

MD and AS-G contributed to the conception and design of the study and implemented the solution. MD performed the statistical analysis. ES and SP also contributed to the conception and design of the study. All authors contributed to the article and approved the submitted version.

## Footnotes

1 As per Section 3, that would be a frame rate of 980 or 1024, depending on the number of cameras.
