# Supplementary material for: Towards practical object detection for weed spraying in precision agriculture
Source: Front Plant Sci. 2023 Nov 3;14:1183277. doi: 10.3389/fpls.2023.1183277 (PMC10657197; doi:10.3389/fpls.2023.1183277)
Supplement: Supplementary file 1 [file DataSheet_1.pdf]

Supplementary Material

ACCURACY AT DIFFERENT GROWTH STAGES

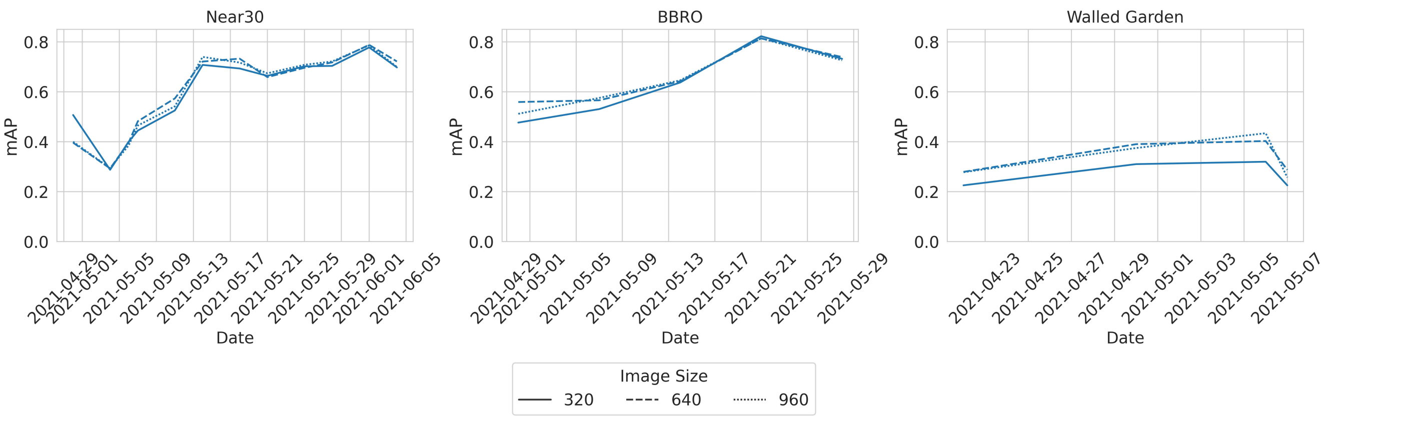

Figure S1. YoloV3: Sugarbeet

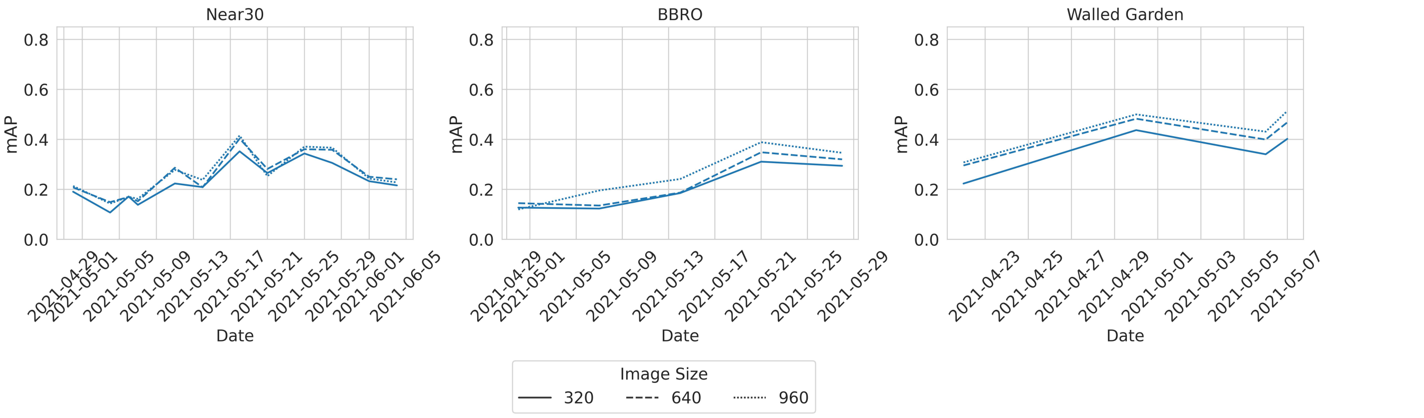

Figure S2. YoloV3: Weed

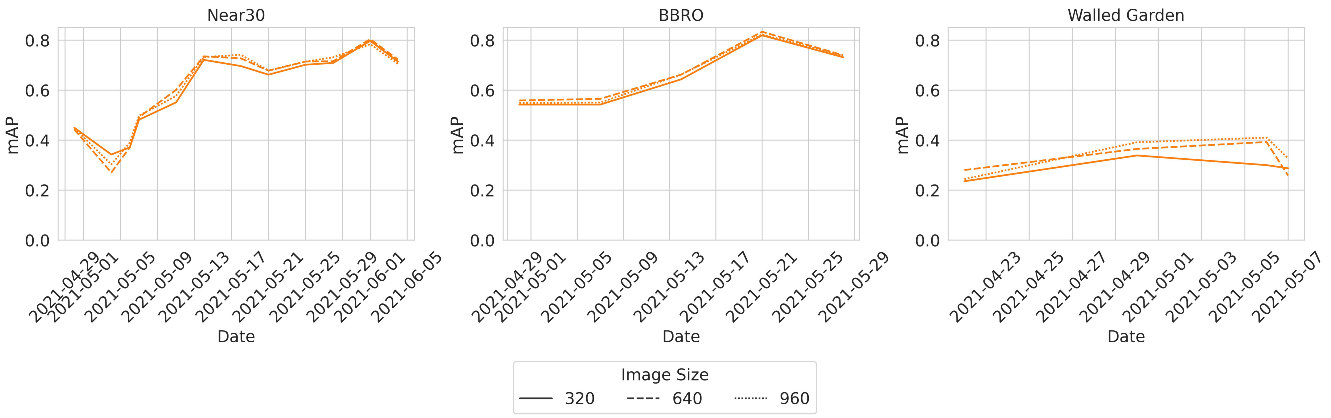

Figure S3. YoloV5s: Sugarbeet

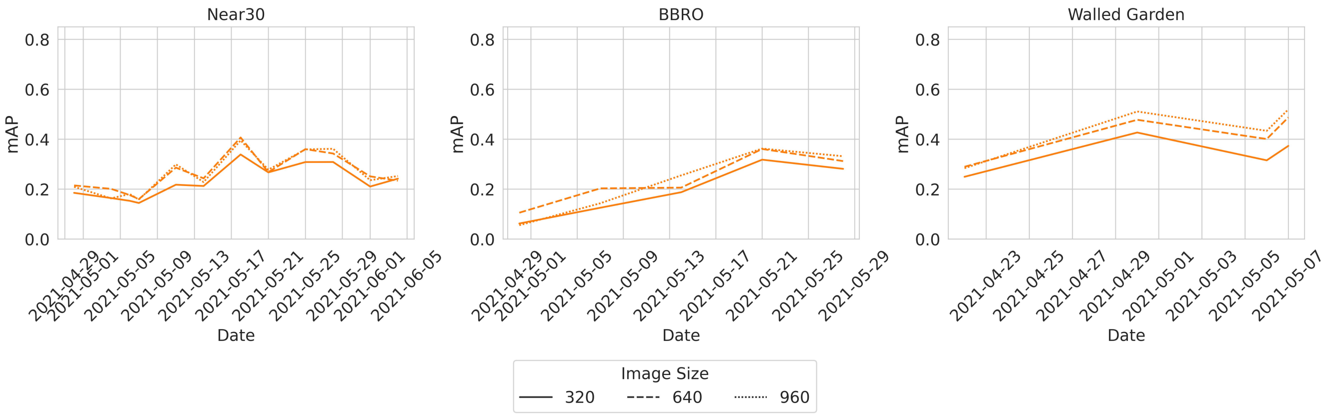

Figure S4. YoloV5s: Weed

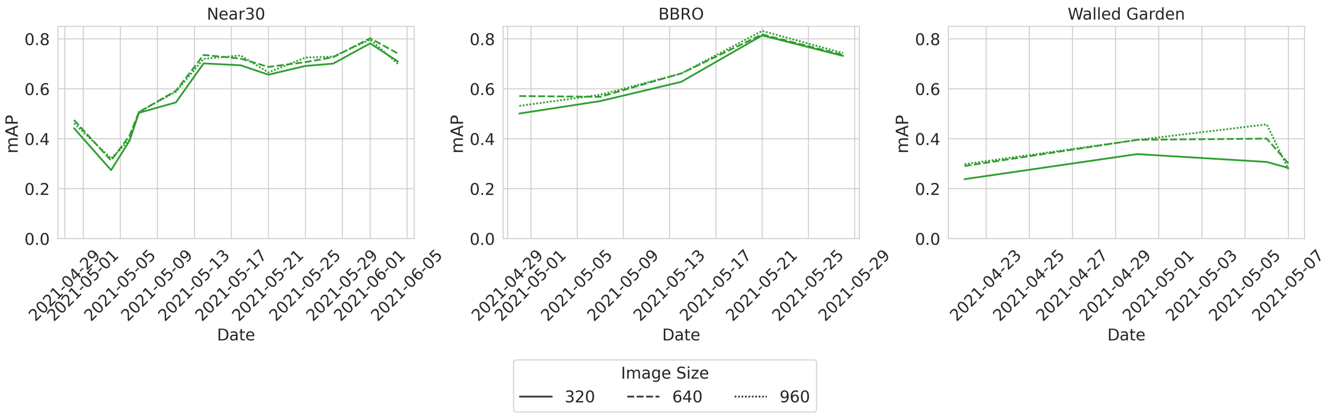

Figure S5. YoloV5m: Sugarbeet

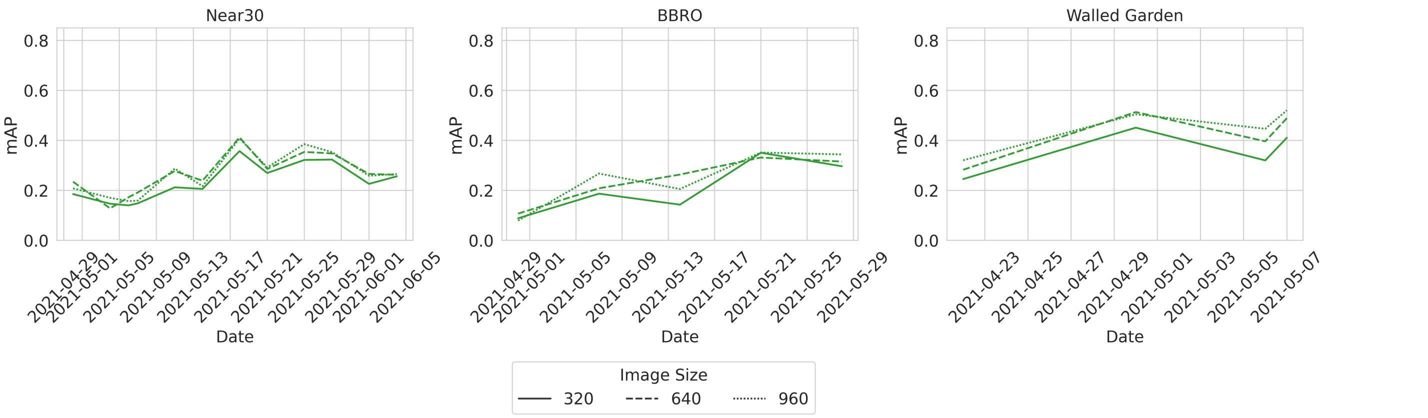

Figure S6. YoloV5m: Weed

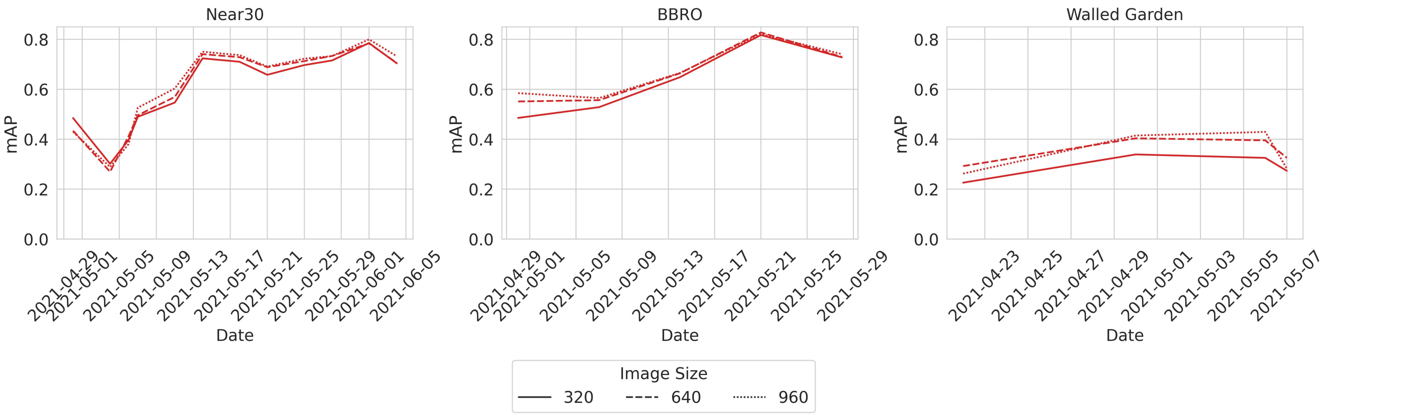

Figure S7. YoloV5l: Sugarbeet

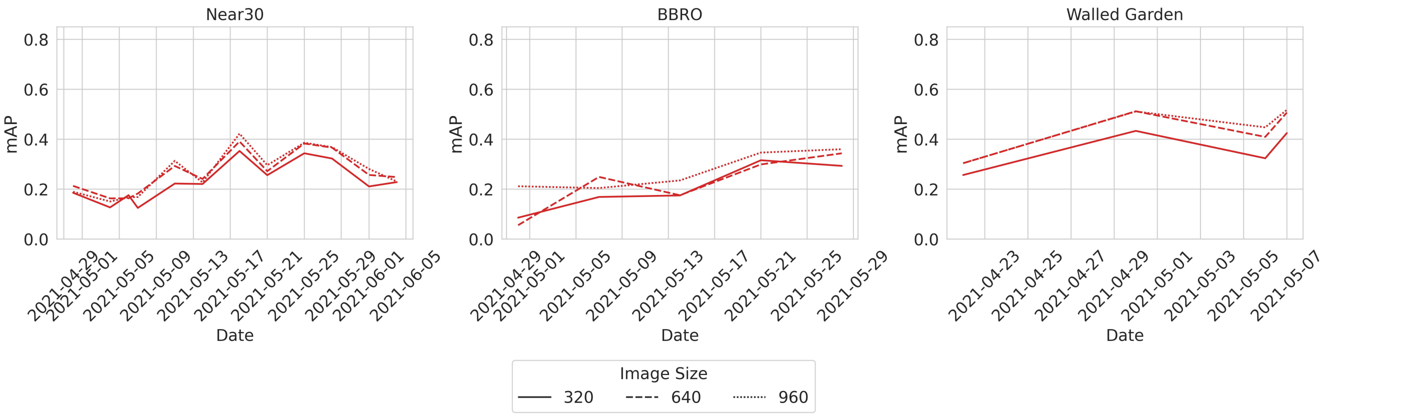

Figure S8. YoloV5l: Weed

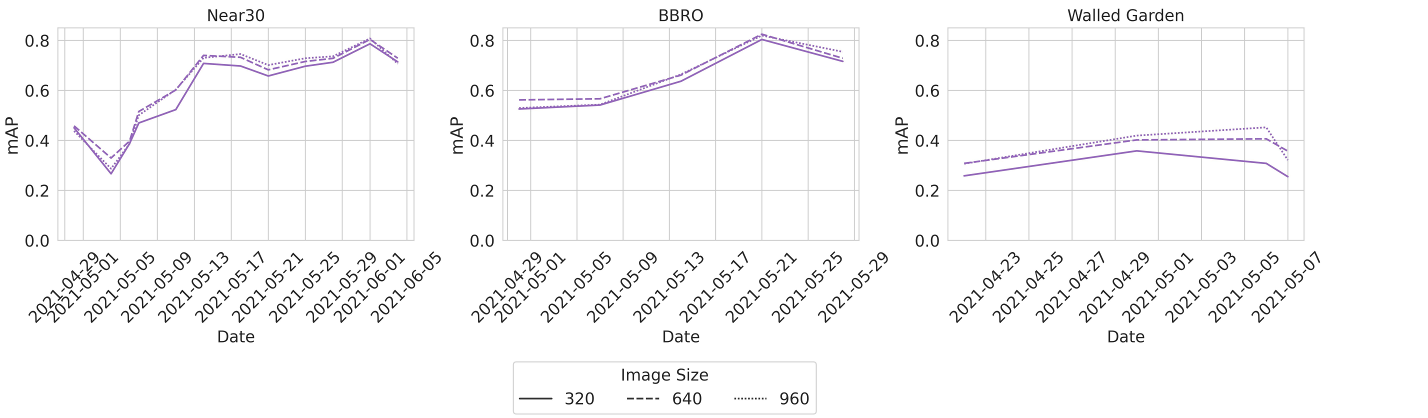

Figure S9. YoloV5x: Sugarbeet

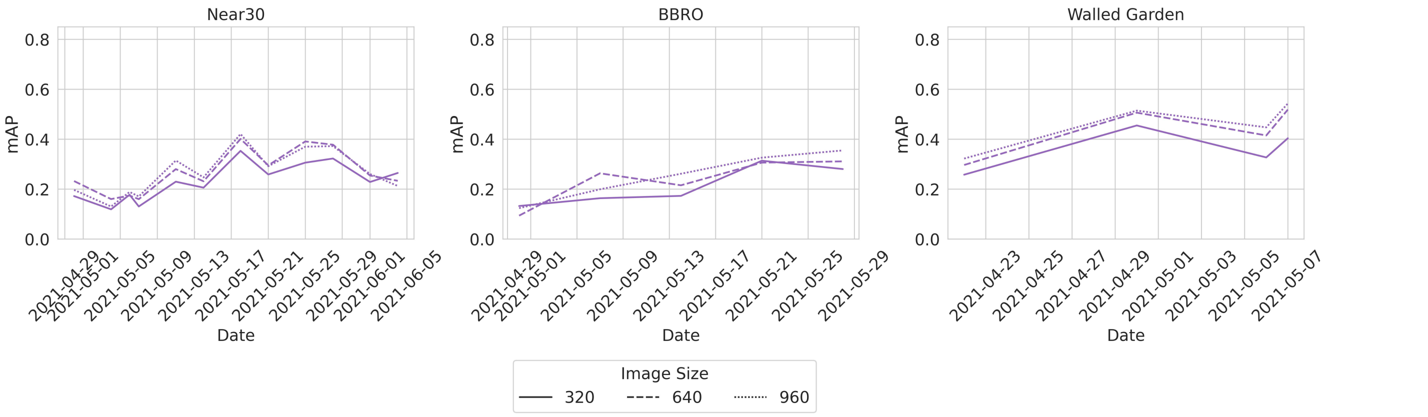

Figure S10. YoloV5x: Weed

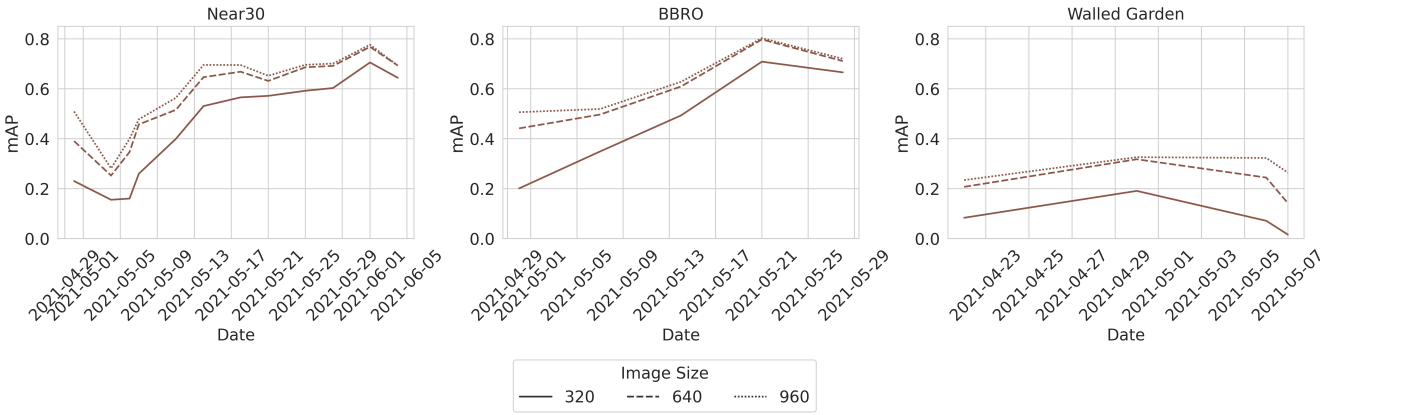

Figure S11. Faster R-CNN ResNet-50-FPN: Sugarbeet

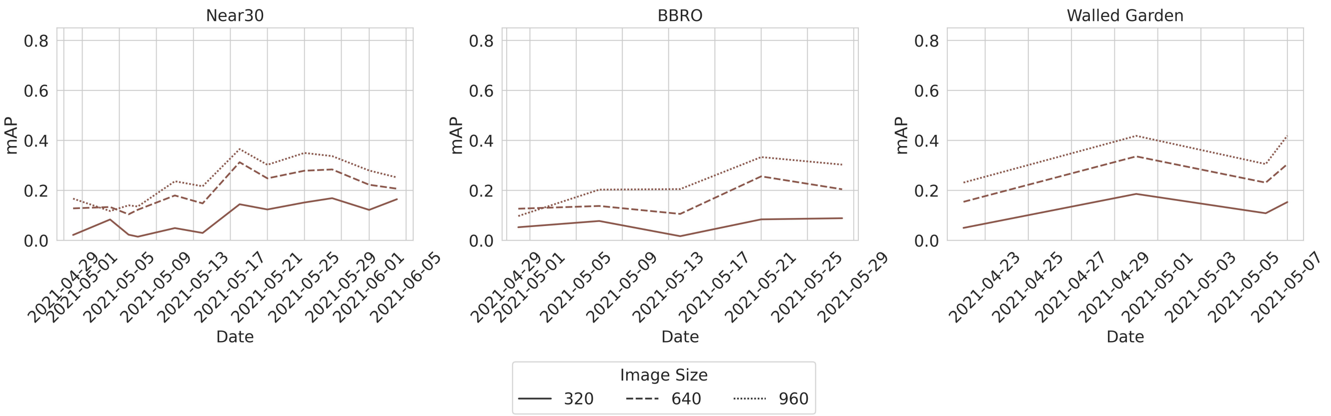

Figure S12. Faster R-CNN ResNet-50-FPN: Weed

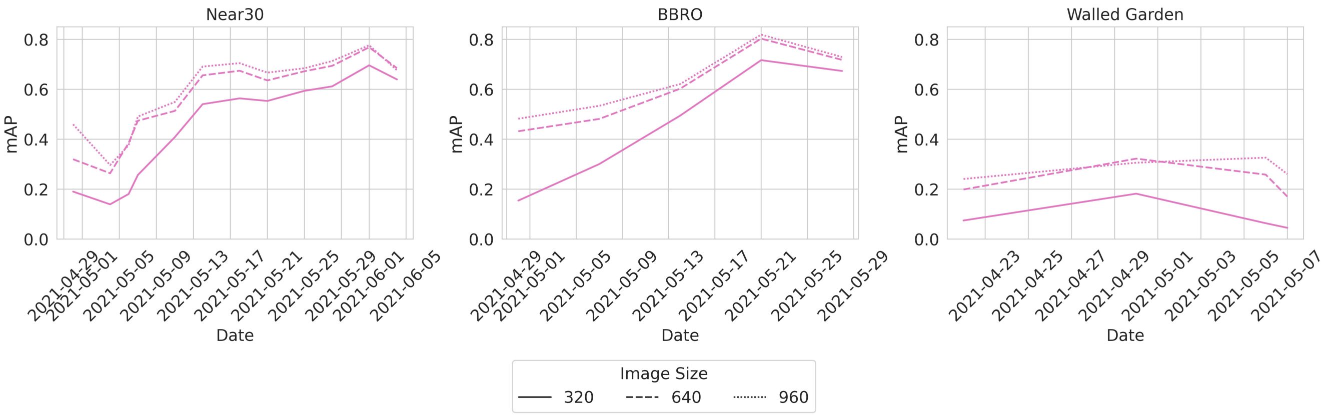

Figure S13. Faster R-CNN ResNet-101-FPN: Sugarbeet

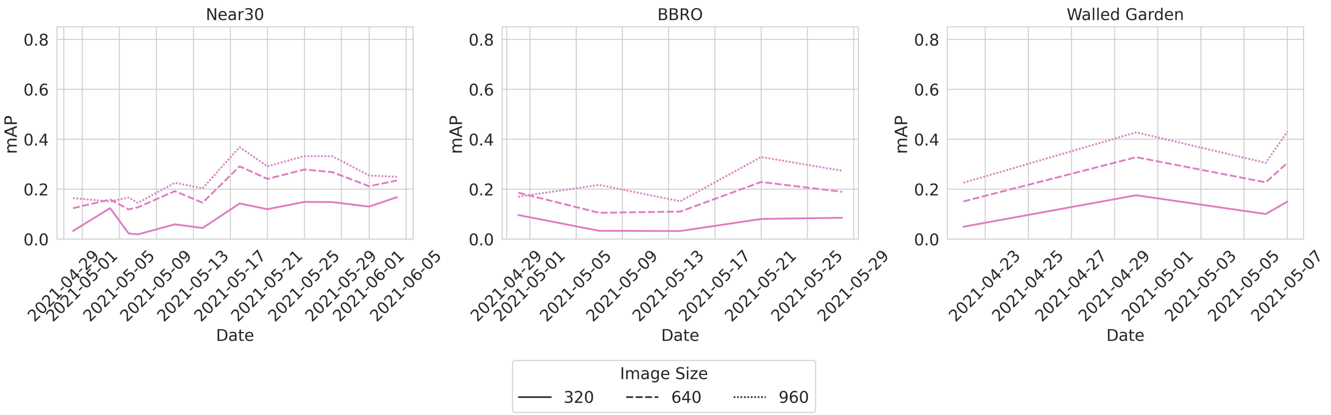

Figure S14. Weed

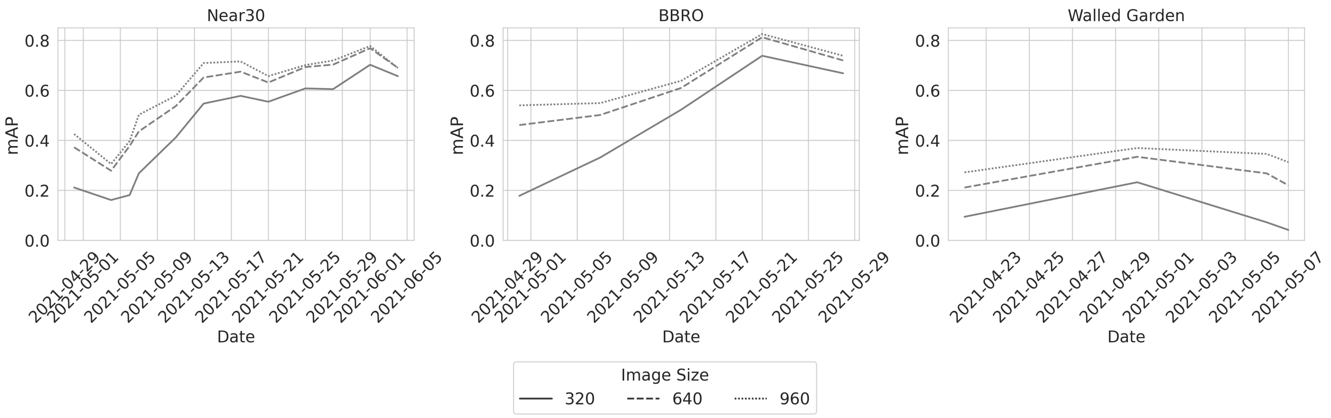

Figure S15. Faster R-CNN ResNeXt-101: Sugarbeet

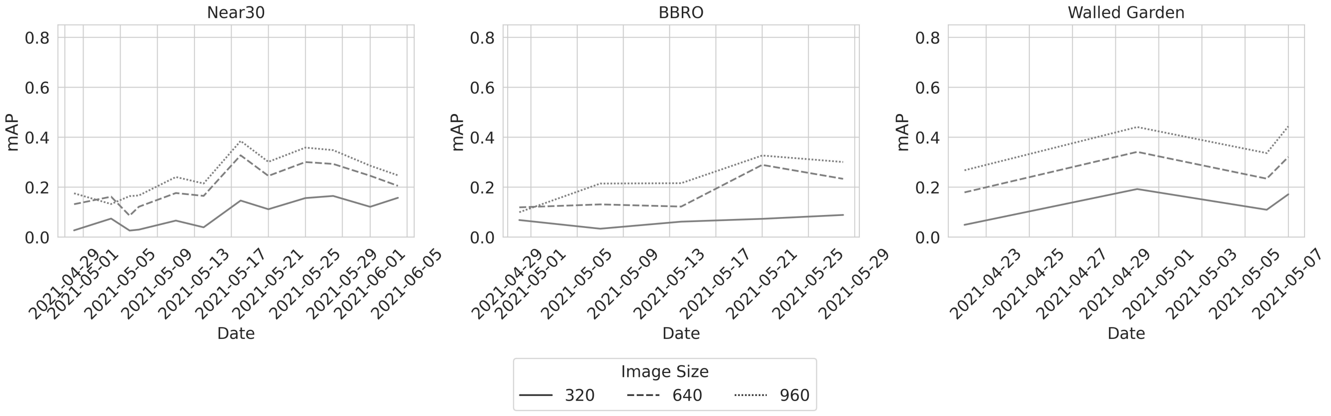

Figure S16. Faster R-CNN ResNeXt-101: Weed
